# Male responsibility and maternal morbidity: a cross-sectional study in two Nigerian states

**DOI:** 10.1186/1472-6963-11-S2-S7

**Published:** 2011-12-21

**Authors:** Neil Andersson, Khalid Omer, Dawn Caldwell, Mohammed Musa Dambam, Ahmed Yahya Maikudi, Bassey Effiong, Edet Ikpi, Etuk Udofia, Amir Khan, Umaira Ansari, Noor Ansari, Candyce Hamel

**Affiliations:** 1 Centro de Investigación de Enfermedades Tropicales, Universidad Autónoma de Guerrero, Calle Pino, El Roble, Acapulco, Mexico; 2 CIET Trust, Calabar and Bauchi, Nigeria; 3 Bauchi State Primary Health Care Development Agency, Nigeria; 4 Bauchi State Ministry of Health, Nigeria; 5 Cross River State Ministry of Health, Nigeria; 6 Institute of Geography, Urban and Regional Planning, University of Peshawar, Pakistan

## Abstract

**Background:**

Nigeria continues to have high rates of maternal morbidity and mortality. This is partly associated with lack of adequate obstetric care, partly with high risks in pregnancy, including heavy work. We examined actionable risk factors and underlying determinants at community level in Bauchi and Cross River States of Nigeria, including several related to male responsibility in pregnancy.

**Method:**

In 2009, field teams visited a stratified (urban/rural) last stage random sample of 180 enumeration areas drawn from the most recent censuses in each of Bauchi and Cross River states. A structured questionnaire administered in face-to-face interviews with women aged 15-49 years documented education, income, recent birth history, knowledge and attitudes related to safe birth, and deliveries in the last three years. Closed questions covered female genital mutilation, intimate partner violence (IPV) in the last year, IPV during the last pregnancy, work during the last pregnancy, and support during pregnancy. The outcome was complications in pregnancy and delivery (eclampsia, sepsis, bleeding) among survivors of childbirth in the last three years. We adjusted bivariate and multivariate analysis for clustering.

**Findings:**

The most consistent and prominent of 28 candidate risk factors and underlying determinants for non-fatal maternal morbidity was intimate partner violence (IPV) during pregnancy (ORa 2.15, 95%CIca 1.43-3.24 in Bauchi and ORa 1.5, 95%CI 1.20-2.03 in Cross River). Other spouse-related factors in the multivariate model included not discussing pregnancy with the spouse and, independently, IPV in the last year. Shortage of food in the last week was a factor in both Bauchi (ORa 1.66, 95%CIca 1.22-2.26) and Cross River (ORa 1.32, 95%CIca 1.15-1.53). Female genital mutilation was a factor among less well to do Bauchi women (ORa 2.1, 95%CIca 1.39-3.17) and all Cross River women (ORa 1.23, 95%CIca 1.1-1.5).

**Interpretation:**

Enhancing clinical protocols and skills can only benefit women in Nigeria and elsewhere. But the violence women experience throughout their lives – genital mutilation, domestic violence, and steep power gradients – is accentuated through pregnancy and childbirth, when women are most vulnerable. IPV especially in pregnancy, women's fear of husbands or partners and not discussing pregnancy are all within men's capacity to change.

## Background

Reputedly one of the highest in the world [1,2], maternal mortality in Nigeria rests on two problems not peculiar to Nigeria, that are easy to state but hard to change. First, as in many countries, maternal health services do not work well. Second, also not specific to Nigeria, maternal deaths follow a life course that puts women at high risk at the time of delivery.

One out of every ten women who attended the Bauchi central referral hospital between 2000-2005 died in relation to childbirth [3]. A review of births over 17 years in neighbouring Plateau State produced much the same figures, indicating the phenomenon is not local [4].

High rates of maternal morbidity and mortality in northern states led some authors to speculate that undervaluing women combines dangerously with harmful traditional medical practices [5]. But studies from the south show very similar pictures of late presentation of morbidity at weak emergency services [6-10]. North and south, the common morbidities are puerperal sepsis, haemorrhage, abortion complications, eclampsia and prolonged obstructed labour. Several studies have focussed on factors underlying these problems. “Poverty” receives several mentions [11-13]; although antenatal and delivery services are officially free at government facilities, in practice almost everyone has some expenditure [14,15]. A study of maternity staff knowledge in two south-western states of Nigeria showed many maternity unit operatives lack knowledge and skills of emergency management [16].

Bauchi in the north of Nigeria is predominantly Islamic; polygamy is common. Cross River is the south-eastern corner of the country, and the main religion is Christian (Evangelical and Catholic). As part of the five-year Nigeria Evidence-based Health System Initiative (NEHSI) [17], the state governments of Bauchi and Cross River nominated maternal outcomes as their first health priority for study. This article results from a bigger process of building evidence-based planning capacity in the health sector, to improve the public health. This analysis examined actionable risk factors and underlying determinants for reduction of maternal morbidity and, as a result, mortality in these two states.

## Methods

A cross-sectional survey in 180 sites in a stratified last stage random sample of the recent census enumeration areas (EAs) in Bauchi and Cross River states. In each state, a panel of 60 sites provided state level representation; in addition, 10 sites in each of three randomly selected focus local government authorities (LGA) in each state provided increased sensitivity of local analysis. Local interviewers identified women who had been pregnant in the previous three years and administered a questionnaire in their language of choice. There was no sub-sampling within the enumeration area, or within households.

State planners chose the focus of the survey, and participated in review of existing data, design of instruments, training of fieldworkers, supervision of fieldwork, analysis and development of emerging policy implications.

A household interview provided household characteristics and a questionnaire for women asked if respondents had given birth in the last three years. For those that had done so, we obtained information on the pregnancy and its outcome, surgical intervention during the delivery and the state of the child. We asked simple direct questions about occurrence of complications: During this last pregnancy did you have fits or convulsions? Did the wound open up afterwards or become infected? Did you develop high fever within six weeks after this delivery? Did you develop foul-smelling discharge from vagina within six weeks after this delivery?

The principal analysis addressed all these complications together, under the hypothesis that positive spouse involvement in the pregnancy would be associated with fewer complications. We repeated the analysis separately for specific morbidities: pre-eclampsia and sepsis. We defined pre-eclampsia as two or more of the following during pregnancy: raised blood pressure, swelling of face or hands, fits/convulsions, or upon testing of their urine, they received information that something was wrong.

Table 1 lists the potential risk factors and underlying determinants covered as direct closed questions. Interviewers asked women about female genital mutilation (FGM) in two questions, one specifically about circumcision and another about removal of genital flesh. They asked women about physical intimate partner violence (IPV) in the last year and, separately, during the last pregnancy (In the last year, have you had violent arguments where your partner beat, kicked or slapped you? During the pregnancy, did your partner beat, kick or slap you?).

**Table 1:** Study population and frequency of maternal knowledge and attitudes in Bauchi and Cross River

	*Bauchi State*		*Cross River State*	
All women interviewed	11486		14268	
Women with pregnancy in the last three years	7870		7759	
Urban	18.2% 1246/7870		30.3% 2617/7759	
Any formal education	22.1% 1719/7834		93.6% 7200/7720	
Married	97.3% 7653/7860		81.0% 6303/7749	
Sufficient food in the last week	89.8% 7072/7845		81.9% 6303/7743	
Remunerated employment	48.1% 3746/7809		59.4% 4544/7749	
Younger age (lower risk for pregnancy)	82.7% 6577/7854		87.0% 6760/7753	
Number of pregnancies (1-3)	48.1% 3786/7749		62.1% 4609/7468	
Female headed household	0.6% 61/6975		10.3% 676/6574	
Non-crowded household (up to two per room)	34.9% 2547/7428		39.4% 3041/7715	

KNOWLEDGE AND ATTITUDES	Weighted	Unweighted	Weighted	Unweighted

Know any danger in pregnancy (1)	53.5%	53.7% 4174/7775	62.8%	63.4% 4909/7746
Know danger signs in childbirth (2)	52.9%	53.6% 4158/7753	47.3%	47.3% 3661/7742
Women should give up heavy work in pregnancy	42.7%	39.0% 3063/7855	37.7%	38.0% 2946/7756
Believe its not okay for pregnant women to smoke cigarettes	80.1%	79.7% 6257/7854	90.2%	89.4% 6924/7746
Believe women without birth problems still need to deliver at a health facility	34.0%	35.9% 2822/7858	71.6%	71.3% 5523/7745
If pregnant next year, would give up heavy work	36.7%	39.1% 2905/7440	48.7%	48.7% 3771/7747
If pregnant next year, would not smoke cigarettes	98.1%	98.2% 7707/7847	99.5%	99.5% 7707/7748
Involved in decisions regarding pregnancy/ childbirth	0.5%	0.5% 41/7821	25.1%	25.6% 1982/7735
Say they were never beaten	95.7%	95.9% 7493/7817	79.7%	80.1% 6145/7673
Say they were not afraid of their husbands	65.9%	65.7% 5137/7821	67.7%	67.5% 5180/7673
No female circumcision or mutilation	90.8%	89.1% 6265/7028	61.7%	61.0% 4702/7707

ABOUT THE LAST PREGNANCY (last three years)	Weighted	Unweighted	Weighted	Unweighted

Spoke about pregnancy primarily with husband	55.4%	56.1% 4151/7399	32.4%	32.9% 2491/7567
Say they were not beaten in pregnancy	97.4%	97.5% 7406/7600	88.8%	89.1% 6558/7358
Reduced workload before 3^rd^ trimester	19.2%	19.1% 1467/7701	52.0%	52.2% 3524/6754
Four or more antenatal checkups	40.4%	40.5% 3012/7446	45.4%	46.4% 3273/7057
Took iron/folate at least one trimester	30.5%	32.6% 2434/7475	44.3%	44.7% 2967/6644
Urine checked at antenatal care	41.9%	39.8% 2955/7432	59.8%	61.8% 4564/7381
Blood pressure checked at antenatal care	59.7%	58.7% 4389/7479	71.0%	73.0% 5382/7372
A qualified person delivered the baby in a health facility	16.4%	15.4% 1170/7590	44.8%	45.0% 3198/7107

1. Any of the following responses: pre-eclampsia, eclampsia, fever, bleeding, lap pain, high blood pressure, cord appears, breech/wrong presentation of baby, vomiting, fits/convulsions, uncontrolled urination, baby movements not felt, weakness, anaemia, jaundice, water coming out, malaria

2. Any of the following responses: malposition, premature labour, prolapse, retained placenta, uncontrolled urine, stillbirth, prolonged/obstructed labour, anaemia, weakness, low blood pressure, sepsis, fever, vaginal cut

The survey occurred from May to November 2009. In each state we standardized training in non-sample sites, training 20-30 fieldworkers over one week. Some 140 interviewers aged 20-35 years worked in 12 teams (one man and two women per team), conducting a general household interview (female interviewer), a husband/spouse interview (male interviewer), and an interview with women who had been pregnant in the last three years (female interviewer).

Teams covered each enumeration area moving radially outwards, excluding no households or women in the households. In a second visit, a smaller team conducted focus group discussions separately with women and men, and visited the government health facilities mentioned by household respondents. There were 180 male and 180 female focus groups; each with 7-10 members with a total participation of 1434 men and 1544 women. The team also reviewed government prenatal and delivery services nearest to each cluster, including issues like access to water, privacy and qualifications of health workers.

Preliminary results provided a template for gender-stratified focus group discussions in each of the 180 clusters. Facilitators asked questions and used standardized prompts and monitors recorded male and female discussions about work during pregnancy, safe pregnancy and safe birth, IPV and FGM.

### Statistical methods

Different operators entered the data twice with validation to minimize keystroke errors. Analysis relied on CIETmap open-source software [18] that offers a user-friendly interface with the now standard statistical programming language R. We weighted all estimates proportional to population within each state, down-weighting the additional sites in the six focus LGAs.

Sequential bivariate analysis allowed examination of the association of each potential risk factor and underlying determinant in turn with maternal morbidity. To verify that associations of risk factors with maternal morbidity could not be explained by any of the general factors (age, sex, crowding, food security, urban/rural or country) we saturated initial multivariate models with the potential risk factors, then stepped down one variable at a time until only significant associations remained. We followed the same procedure for the Mantel Haenszel procedure and for GEE which accessed Zelig [19], applying an exchangeable correlation structure (logit.gee model, 1000 simulations). We report the adjusted Odds Ratio (ORa) and cluster-adjusted confidence intervals (CIca) using a robust variance estimator to weight the confidence interval around the Mantel Haenszel Odds Ratio for cluster-correlated data [20,21].

The sample represents only those present at the time of the fieldwork; we have no information on why others were absent. Very few women declined to take the survey and we made no effort to persuade them to do so. More women in Bauchi than in Cross River declined to answer questions about genital mutilation and domestic violence. Clustering effects were different in Bauchi, where polygamy is more widespread and it was more common to have multiple women who gave birth in a single household.

### Ethics

In Bauchi, the Ethics Review Committee of the State Ministry of Health provided general approval in April 2009. The Cross River State Research Ethics Committee approved the methods and survey instruments on 28 August 2009, and the qualitative procedures in January 2010.

## Results

Female interviewers administered questionnaires to 25,745 women of a possible 30,918 in the two states; 1.2% declined the interview (345 or 1.8% in Cross River and 37 or 0.3% in Bauchi); a further 15% were not available at the time of the visit (4,213 or 22.2% in Cross River, where more women have formal employment, and 429 or 3.6% in Bauchi). A total of 15,621 women had given birth (7,759 in Cross River and 7,862 in Bauchi) in the last three years.

Table 1 lists the frequency of household characteristics, male knowledge and attitudes, antenatal care, work during pregnancy, IPV and FGM, and female knowledge, attitudes, intentions, and agency. One third lived in urban areas in Cross River, one half of that proportion in Bauchi. Nearly all Cross River women had formal education compared with one in every four Bauchi women.

Reports of pre-eclampsia and eclampsia were comparable in Bauchi (10.3% weighted value of 842/7684) and Cross River (13.0% weighted value of 973/7178). However, post-partum sepsis was much more common in Cross River (30.6% weighted value of 2223/7176), compared with 5.6% (weighted value of 473/7724 in Bauchi). The principal analysis combined pre-eclampsia, sepsis and other complications including excessive bleeding and convulsions as maternal morbidity related to pregnancy, delivery or post delivery: 17.8% of women in Bauchi and 43.9% in Cross River reported one of these.

Table 2 shows the bivariate associations between all potential risk factors and underlying determinants studied and maternal morbidity, indicating a number of promising associations. In addition, in both states, postnatal visits were more common among women who reduced work before the third trimester of pregnancy, who had more antenatal check-ups, who delivered at the health centre, who had healthy attitudes to smoking in pregnancy and who were more likely to know of danger signs in pregnancy. In general, women receiving postnatal visits were better off: they were more likely to have some education, less likely to complain of food insecurity and less likely to live in crowded households.

**Table 2:** Bivariate associations between maternal morbidity and potential risk factors

Variable	Bauchi			Cross River		
	
	With problem with factor	With problem without factor	OR (95% CIca)	With problem with factor	With problem without factor	OR (95% CIca)
Urban	22.5% 274/1219	18.1% 1155/6378	1.31 (1.00-1.72)	45.7% 1093/2393	43.9% 2097/4777	1.07 (0.90-1.28)
Any formal education	22.5% 374/1660	17.7% 1046/5903	1.35 (1.10-1.66)	44.8% 2971/6637	41.5% 290/496	1.14 (0.94-1.39)
Married	18.7% 1382/7393	22.6% 44/195	0.79 (0.57-1.09)	44.2% 2608/5896	45.6% 577/1264	0.94 (0.82-1.09)
Food security in last week	18.2% 1239/6824	24.8% 186/749	0.67 (0.55-0.81)	42.9% 2502/5835	51.6% 682/1321	0.70 (0.63-0.78)
Remunerated employment	20.7% 753/3629	17.1% 668/3909	1.27 (1.08-1.50)	45.9% 1931/4207	42.5% 1254/2954	1.15 (1.02-1.30)
Low risk age for pregnancy (18-35 yrs)	18.7% 1186/6356	19.7% 242/1226	0.93 (0.79-1.10)	45.1% 2832/6274	40.0% 356/890	1.23 (1.06-1.43)
Times pregnant (1-3 pregnancies)	16.5% 605/3658	20.9% 798/3827	0.75 (0.65-0.87)	44.4% 1945/4385	44.7% 1233/2761	0.99 (0.90-1.08)
Female headed household	25.4% 15/59	18.8% 1251/6672	1.48 (0.73-3.00)	50.6% 159/314	48% 1479/3079	1.16 (0.94-1.42)
Non-crowded households (2/room or less)	19.9% 487/2451	18.4% 870/4719	1.10 (0.94-1.28)	46.7% 652/1395	49.1% 1229/2501	0.86 (0.76-0.97)
Know any danger in pregnancy	20.1% 817/4065	17.3% 596/3441	1.20 (1.06-1.36)	44.5% 2035/4577	44.6% 1151/2583	1.00 (0.90-1.10)
Know danger signs in childbirth	20.2% 817/4042	17.2% 592/3445	1.22 (1.07-1.39)	47.2% 1600/3389	42.0% 1583/3765	1.23 (1.10-1.38)
Believe women should give up heavy work in pregnancy	17.4% 511/2938	19.7% 914/4646	0.86 (0.75-0.98)	44.7% 1217/2720	44.3% 2476/4448	1.02 (0.91-1.14)
Believe it's not okay for pregnant women to smoke cigarettes	18.2% 1100/6046	21.2% 326/1536	0.83 (0.70-0.98)	45.8% 3467/6393	34.1% 261/765	1.63 (1.36-1.95)
Believe women without birth problems still need to deliver at a health facility	19.2% 526/2742	18.6% 899/4843	1.04 (0.89-1.21)	45.2% 2305/5105	42.8% 879/2052	1.10 (0.99-1.22)
Intention: If pregnant next year, would give up heavy work	18.4% 515/2800	19.4% 849/4381	0.94 (0.83-1.06)	45.2% 1573/3477	43.8% 1614/3681	1.06 (0.94-1.19)
Intention: If pregnant next year, would not smoke cigarettes	18.6% 1387/7443	26.3% 35/133	0.64 (0.38-1.07)	44.5% 3170/7120	33.3% (26/39)	1.61 (0.77-3.33)
Involved in decisions regarding pregnancy/childbirth	45.0% 18/40	18.7% 1404/7514	3.56 (1.98-6.39)	46.9% 849/1811	43.7% 2335/5340	1.14 (1.00-1.28)
Spoke about pregnancy primarily with husband	20.1% 802/3956	17.3% 546/3156	1.20 (1.06-1.37)	42.8% 1006/2349	45.4% 2115/4654	0.90 (0.80-1.01)
Say they were not ever beaten	18.4% 1332/7236	28.2% 89/316	0.58 (0.41-0.81)	42.4% 2414/5695	53.1% 745/1403	0.65 (0.58-0.73)
Say they were not beaten in pregnancy	18.9% 1357/7183	24.6% 46/187	0.71 (0.48-1.07)	43.3% 2732/6307	53.8% 415/772	0.66 (0.56-0.77)
Say they were not afraid of their husbands	18.6% 933/5018	19.1% 486/2538	0.96 (0.81-1.15)	43.0% 2062/4795	47.6% 1096/2304	0.83 (0.73-0.94)
Reduced workload before third trimester	22.5% 318/1416	18.0% 1084/6023	1.32 (1.10-1.58)	42.7% 1424/3334	45.9% 1444/3149	0.88 (0.79-0.98)
Had four or more antenatal check-ups	22.0% 650/2952	16.9% 726/4300	1.39 (1.19-1.62)	44.1% 1418/3218	45.4% 1628/3582	0.95 (0.84-1.06)
Took iron-folate at least one trimester	20.8% 496/2385	17.5% 850/4847	1.23 (1.06-1.44)	44.8% 1300/2904	44.8% 1155/3470	1.00 (0.90-1.11)
Urine checked at antenatal clinic	21.4% 619/2891	17.5% 758/4324	1.28 (1.07-1.54)	43.0% 1890/4400	47.1% 1271/2700	0.85 (0.74-0.96)
Blood pressure checked at antenatal clinic	20.8% 892/4294	16.6% 492/2965	1.32 (1.09-1.60)	43.4% 2256/5204	47.6% 898/1885	0.84 (0.72-0.98)
Qualified person at delivery at health facility	28.0% 321/1147	16.8% 1046/6211	1.92 (1.59-2.31)	41.8% 1322/3162	46.8% 1816/3882	0.82 (0.72-0.93)
Did not experience female circumcision	18.5% 1121/6074	23.8% 176/741	0.73 (0.58-0.90)	42.6% 1851/4346	47.6% 1323/2779	0.82 (0.73-0.91)

Table 3 shows the final multivariate models for all complications combined. In Bauchi, initial analysis of non-fatal maternal morbidity (pre-eclampsia, sepsis, excessive haemorrhage) showed marked heterogeneity between the minority of women who had a health check up after delivery and the majority who did not. Among those who received a check up, two factors remained in the final model: FGM (ORa 2.10 95%CIca 1.39-3.17) and four or more pregnancies (ORa 1.48, 95%CIca 1.15-1.90). FGM remained in both models in Cross River.

**Table 3:** Multivariate analysis of non-fatal maternal morbidity risk factors

	OR unadjusted	Mantel Haenszel analysis with cluster adjustment		GEE with exchangeable correlation matrix	
	
		**OR**^1^**adjusted**	Cluster adjusted 95%CI	OR^2^	Robust 95%CI
**Bauchi**				n	

With check-up after delivery		n=1137		n=1307	

FGM	2.13	2.1	1.39-3.17	1.93	1.35-2.77
4+ pregnancies	1.49	1.48	1.15-1.90	1.46	1.14-1.87

No check-up after delivery		n=5196		n=6005	

Did not speak primarily with husband	1.35	1.41	1.21-1.67	ns	
Physical IPV in pregnancy	2.15	2.15	1.43-3.24	2.12	1.41-3.18
Unqualified birth attendant	1.59	1.61	1.23-2.13	1.48	1.17-1.86
Insufficient food last week	1.68	1.66	1.22-2.26	1.46	1.14-1.86
4+ pregnancies	1.26	1.24	1.05-1.48	1.28	1.08-1.51
Less than 4 ANC check-ups	1.24	ns	ns	1.24	1.05-1.46

**Cross River**					

With check-up after delivery		n=2201		n=2307	

IPV last year	1.6	1.56	1.20-2.03	1.58	1.24-2.02
FGM	1.28	1.29	1.10-1.51	1.3	1.12-1.50
Did not speak primarily with husband	1.28	1.31	1.11-1.55	1.25	1.07-1.48
Crowded home (>2/room)	1.27	1.27	1.07-1.51	1.27	1.07-1.51
Formal employment	1.25	1.22	1.01-1.49	ns	ns

No check-up after delivery		n=4221		n=4856	

IPV last year	1.5	1.43	1.24-1.65	1.3	1.10-1.54
FGM	1.2	1.19	1.03-1.37	ns	ns
Physical IPV in pregnancy		ns	ns	1.37	1.09-1.74
Unqualified birth attendant	1.33	1.22	1.05-1.41	ns	ns
Insufficient food last week	1.42	1.32	1.15-1.53	1.28	1.10-1.48
Aged 18-35 years	1.32	1.3	1.06-1.59	1.39	1.14-1.71
Did not reduce workload	1.26	1.21	1.08-1.35	1.14	1.02-1.27

**^1^**Odds Ratio for the association between the variable and maternal morbidity, adjusted for all other variables in the final multivariate model. The initial model was based on the covariates in Table 2

**^2^**An identical modelling process served for GEE

ns = not statistically significant at the 5% level

Physical IPV during pregnancy showed the strongest association with maternal morbidity in all multivariate models except the small group of Bauchi women who had home visits after delivery. This prominent role remained unchanged when we repeated the analysis using GEE.

Among women who had no home visit after delivery, those who had an unqualified birth attendant (most often to a traditional midwife without government approved training, less often to a neighbour or a family member) were more likely to have complications in both states.

We constructed a compound variable of factors related to the role of a husband or partner in the final model: IPV in pregnancy, IPV in the last year, and report that women had not discussed pregnancy with their husband or partner. Women with all three directly husband-related factors were much more likely to report a pregnancy or birth complication than women who had none, one or two of these factors (ORa 2.39, 95%CIca 1.96-2.92, RD 0.207, 222/432 women with all three and 4,397/14,335 who did not). This association was not explained by any of the factors we could take into account in this study.

Table 4 shows the final models for risk factors for pre-eclampsia and sepsis. Both initial models included the risk factors shown in Table 2. As associations with pre-eclampsia were not significantly different in Bauchi and Cross River, we combined the states for analysis of pre-eclampsia. Four variables showed independent associations after adjusting for the others: IPV in the last year, IPV during the pregnancy in question, rural residence and FGM.

In the case of sepsis, the variable “state” modified most bivariate measured associations, so we developed a separate multivariate model for Bauchi and Cross River. In Bauchi, sepsis was independently associated with IPV in the last year, IPV in the last pregnancy, perception of being cared for in pregnancy, age of the mother (younger women more likely to suffer sepsis) and FGM (Table 4). In Cross River, only two variables remained in the final model, IPV in the last year and perception of being cared for during the pregnancy.

**Table 4:** Multivariate analysis of risk factors for pre-eclampsia and sepsis

			Cross River	
	
	**OR**^1^**adjusted**	Cluster adjusted 95%CI	**OR**^1^**adjusted**	Cluster adjusted 95%CI
**Pre-eclampsia**	Bauchi and Cross River			

IPV last year	1.39	1.17-1.65		
IPV during this pregnancy	1.27	1.01-1.58		
Rural residence	1.38	1.17-1.62		
FGM	1.15	1.02-1.29		

**Sepsis**				

	Bauchi n=6992		Cross River n=7671	

IPV in last year	1.4	1.22-1.61	2.29	1.42-3.68
IPV in last pregnancy	1.27	1.06-1.53		
Did not feel cared for during pregnancy	1.35	1.15-1.59	1.65	1.21-2.24
Age over 30 years	1.18	1.03-1.34		
FGM	1.21	1.08-1.40		

**^1^** Odds Ratio for the association between the variable and maternal morbidity, adjusted for all other variables in the final multivariate model. The initial model was based on the covariates in Table 2

Table 5 shows the low levels of male knowledge of pregnancy and delivery, and the high level of good intentions about maternal risks.

**Table 5:** Male knowledge and attitudes about pregnancy and childbirth in Bauchi and Cross River States, Nigeria

	*Bauchi State*		*Cross River State*	
Men interviewed	2433		2623	

	Weighted	Unweighted	Weighted	Unweighted

Know any danger in pregnancy (1)	31.1%	31.0% 706/2276	37.1%	35.9% 874/2432
Know danger signs in childbirth (2)	44.7%	41.7% 947/2273	38.1%	37.8% 921/2435
Agree male health workers can do antenatal checkups	28.6%	30.4% 714/2352	82.2%	82.1% 2033/2477
Agree male health worker can do deliveries	21.9%	23.6% 554/2351	76.1%	76.1% 1886/2477
Agree it's good pregnant women get together to talk	94.2%	95.5% 2243/2350	94.3%	94.4% 2331/2469
Agree that women should give up heavy work in pregnancy	45.1%	40.2% 944/2351	44.4%	44.3% 1099/2479
Agree it's not okay for pregnant women to smoke cigarettes	77.8%	80.3% 1888/2352	90.6%	90.7% 2249/2480
Agree women with birth complications should deliver at a health facility	98.3%	98.5% 2313/2348	99.0%	98.9% 2451/2478
Believe women sometimes deserve to be beaten	9.8%	8.8% 205/2339	29.3%	29.2% 723/2475
Believe “in my culture, it is acceptable for a man to beat his wife”	7.5%	6.9% 161/2342	20.8%	20.5% 506/2472
Believe violence between a man and a women is private and others should not interfere	46.3%	45.6% 1069/2344	73.9%	74.8% 1852/2475
Willing to take time to accompany wife if she had danger in childbirth	70.3%	68.8% 1531/2226	97.6%	97.4% 2321/2382
Willing to spend on transport for wife if she had danger in childbirth	96.6%	96.6% 2209/2288	86.1%	87.2% 2118/2429
In future, if wife had danger in childbirth, would take her to health facility	96.2%	96.9% 2203/2273	92.7%	93.5% 2279/2438

Main source of information on pregnancy and childbirth				

Don't get any	4.1%	4.2% 96/2316	2.4%	2.6% 63/2468
Family/friends	26.5%	26.3% 610/2316	23.6%	24.2% 596/2468
Media	43.3%	39.1% 905/2316	17.0%	16.9% 417/2468
Health worker	26.0%	30.3% 702/2316	55.3%	54.5% 1344/2468

1. Any of the following responses: pre-eclampsia, eclampsia, fever, bleeding, lap pain, high blood pressure, cord appears, breech/wrong presentation of baby, vomiting, fits/convulsions, uncontrolled urination, baby movements not felt, weakness, anaemia, jaundice, water coming out, malaria

2. Any of the following responses: malposition, premature labour, prolapse, retained placenta, uncontrolled urine, stillbirth, prolonged/obstructed labour, anaemia, weakness, low blood pressure, sepsis, fever, vaginal cut

Male focus groups discussed what men consider when deciding where a woman should deliver her child. Almost all groups recognized a need for skilled birth attendance, and almost all raised economic considerations in taking advantage of this where it was available. “The man considers the weight of his pocket before deciding where to take the woman for delivery”.

Few of the 180 male focus groups saw men as the cause of IPV; nearly all concluded that IPV could be avoided if women prayed, were obedient and patient, and never refused sex. Asked how IPV could be avoided, several groups suggested increasing women's incomes. The focus groups were uniform in the belief that IPV is a private matter, reporting of IPV bringing shame, disgrace and “greater divisions”. In Cross River, men quoted the Bible (“What God has joined together, let no man put asunder”) as the reason for not reporting IPV. In both states, men gave prominence to community leaders and religious leaders to stop the violence. Despite the strong and uniform belief that IPV is a private matter, many male groups were in favour of locally administered punitive schemes, typically a fine for beating one's wife being a goat, or cash ranging from N500 to N10,000 (US$4-70). Asked what men could do themselves, most groups felt they had the power to stop IPV, “As heads of the households, we can do it”.

A clear theme in the 180 female focus groups was self-blame for the IPV (“strong mouth”, disobedient, demanding or refusing sex). Some concluded that men were “naturally violent so there is nothing you can do”. Others said pregnancy was a cause of violence as it made women irritable and too tired to have sex. They saw marital infidelity as a common cause, whether the woman or man was cheating. Across all regions of both states, women saw money as a major cause. According to women in Cross River, “the Bible says that the wife does not have rights over her body, so we should submit our body to our husbands...” and “the Bible says that God created the woman out of Adam’s rib, the woman should be under the man and should be humble to the man’s relatives to avoid being beaten by the man.” In Cross River, women saw IPV as a family matter, to be resolved at home. In clear contrast, no women's focus group in Bauchi reported this view.

## Discussion

Within the constraints of a cross-sectional survey of childbirth survivors, IPV during pregnancy and history of IPV in the last year were the most prominent risk factors or underlying determinants for maternal morbidity in both Bauchi and Cross River.

This study relies on self-reporting of morbidity by survivors of childbirth. Reports of morbidity were quite different between the two states, compatible with different levels of health literacy and the marked differences in women's education between the states. We reduced the effect of this by analysing the two states separately and combining types of maternal morbidity. Despite this reporting difference, spouse-related factors (IPV in the last year, IPV in pregnancy, did not discuss pregnancy primarily with husband) were prominent in both states.

Analysis of individual morbidities (pre-eclampsia and sepsis) showed very much the same picture.

We were initially surprised that women in Cross River reported more delivery complications than women in Bauchi, although many more in River Cross benefited from institutional deliveries. Women in Cross River were also more likely to report IPV and FGM. We do not interpret this to mean these risks are actually higher in Cross River, rather that those women who suffered them were less likely to report them if they were less educated and had less contact with health services. Female education levels were much lower and far fewer women had institutional deliveries in Bauchi than in Cross River. Although we have no detailed information on this from the questionnaires, it is plausible that less educated women considered these problems normal or, having survived, inconsequential. There may also be different social imperatives, interpretations of family pride, between Cross River and Bauchi. This likely under-reporting of complications among women who are at highest risk invalidates unstratified comparison of rates in Bauchi and Cross River. However, it is difficult to compare rates among educated women who have access to care, because there are so few such women in Bauchi.

Associations with maternal morbidity differed between the advantaged women receiving a postnatal home visit, and the majority of women who did not. We offset this by analyzing the groups separately. In both states, those who received a home visit were evidently better off and more engaged with the health services; their risk factors in Bauchi were physical, FGM and multiple (four or more) pregnancies. In all other groups, IPV and socio-economic factors were prominent.

This was a cross-sectional study, with all the usual issues of direction of causality of even the strongest associations. Some spouse-related factors not specific to the pregnancy (IPV in the last year) might be causally related to maternal outcomes or they might result from the maternal outcome or something else shared with the maternal outcome that we neglected to study. It seems likely that the IPV reported during pregnancy preceded the maternal morbidity; it is also possible that women who suffered complications remembered violence differently. Either way, the associations are a cause for concern for pregnant women.

Husband related risk factors and underlying determinants affect many women. Some 45% of women in Bauchi and 68% in Cross River did not say they discussed their pregnancy primarily with their husbands or partners. Only one in five women in Bauchi and one half in Cross River reduced their workload before the third trimester (Table 1). Related to patriarchy though not narrowly to the behaviour of the husband during pregnancy [22], at least one in every ten Bauchi women and four in ten Cross River women entered reproductive life with mutilated genitals.

The protective association between maternal morbidity and the birth attendance by a qualified midwife in both Bauchi and Cross River (Tables 3 and 4) is especially important given the low level of participation of women in decisions about where the birth should be attended. In Bauchi, only 15.6% of women we interviewed had delivered in a health facility. Although the household survey showed good intentions if little knowledge among male respondents (Table 5), focus groups with men showed a prominent belief that maternal outcomes were a question for health services.

The levels of IPV we detected in the two states are within the range of other studies of IPV in pregnancy in Nigeria [23-25]. Associations of maternal morbidity with IPV are well documented in eclampsia [26,27], pre-term delivery [28,29], mental health [30,31], alcohol and tobacco use [32], and health seeking behaviour [33-35]. Little is known of the mechanisms underlying these associations with IPV, and our study is not the design to add major insights. Depression [31,36] and stress [30] are plausible intermediaries. Whatever the mechanism, it is clear that men play an important if not pivotal role – and it is a role they can change. The few calls for men to play a role in favour of prevention of maternal mortality [37-39] have not been accompanied by larger scale programmes that address maternal morbidity through working with men.

## Conclusions

In this study as in others in other places, violence against women is strongly associated with maternal morbidity. Reduction of these risk factors and underlying determinants involves spouses, independent of the health services. The sample represents the northern Bauchi state and Cross River in the south east of Nigeria. High levels of FGM, maternal mortality and pregnancy complications in the predominantly Christian south contradict any notion that these are limited to the predominantly Muslim north. Across these widely different settings and consistent with existing literature, male responsibility is important in maternal mortality.

Our focus on men in prevention of maternal morbidity does not detract from the good reasons to increase coverage with antenatal care and access to health facilities. Enhancing the clinical protocols and skills of health workers can only be of benefit to women in Nigeria and elsewhere. But, with prominence of men in the strongest risk factors for and underlying determinants of maternal morbidity, efforts to increase coverage and quality of obstetric care should take care not to bolster the male belief that maternal health is not their responsibility.

Our study opens another arena for reduction of maternal morbidity, with men as possible agents for change. The violence women experience throughout their lives – genital mutilation, domestic violence, and steep power gradients – is accentuated through pregnancy and childbirth, when women are most vulnerable. IPV especially in pregnancy, women's fear of husbands or partners and being able to discuss pregnancy with their husbands or partners are all within the male domain.

## Competing interests

The authors declare they have no competing interests.
